# Modulatory Properties of *Aloe secundiflora’s* Methanolic Extracts on Targeted Genes in Colorectal Cancer Management

**DOI:** 10.3390/cancers15205002

**Published:** 2023-10-16

**Authors:** John M. Macharia, Timea Varjas, Ruth W. Mwangi, Zsolt Káposztás, Nóra Rozmann, Márton Pintér, Isabel N. Wagara, Bence L. Raposa

**Affiliations:** 1 Doctoral School of Health Sciences, Faculty of Health Science, University of Pẻcs, 7621 Pẻcs, Hungary; 2 Department of Public Health Medicine, Medical School, University of Pẻcs, 7621 Pẻcs, Hungary; 3 Department of Vegetable and Mushroom Growing, Hungarian University of Agriculture and Life Sciences, 1118 Budapest, Hungary; 4 Department of Biological Sciences, Egerton University, Nakuru P.O. Box 3366-20100, Kenya; 5 Faculty of Health Sciences, University of Pécs, 7621 Pécs, Hungarybence.raposa@etk.pte.hu (B.L.R.)

**Keywords:** molecular mechanism, colorectal cancer, genome modulatory pathways, tumor microenvironment, phytotherapeutic effects

## Abstract

**Simple Summary:**

This study focused on understanding the potential use of *Aloe secundiflora* (AS) extracts in managing colorectal cancer (CRC). As colon tumors present complex challenges, the research aimed to assess how the AS methanolic extracts impacted the expression of specific genes related to CRC, namely *CASPS9*, *5-LOX*, *Bcl2*, *Bcl-xL*, and *COX-2.* The results demonstrated that the AS extracts, when applied to CRC cell lines, effectively upregulated *CASPS9* expression, promoting apoptosis in a dose-dependent manner. Simultaneously, the extracts downregulated the expressions of *5-LOX*, *Bcl2*, and *Bcl-xL*, crucial in curbing cancer progression. The study suggests that using AS extracts and methanol as an extraction solvent could be beneficial in managing CRC. Furthermore, the researchers recommend exploring the specific metabolites in AS involved in these pathways to better understand how they impede the development and spread of CRC. This research provides promising insights into potential natural treatments for colorectal cancer, offering hope for improved therapies in the future.

**Abstract:**

Colon tumors have a very complicated and poorly understood pathogenesis. Plant-based organic compounds might provide a novel source for cancer treatment with a sufficient novel mode of action. The objective of this study was to analyze and evaluate the efficacy of *Aloe secundiflora’s* (AS) methanolic extracts on the expression of *CASPS9*, *5-LOX*, *Bcl2*, *Bcl-xL*, and *COX-2* in colorectal cancer (CRC) management. Caco-2 cell lines were used in the experimental study. In the serial exhaustive extraction (SEE) method, methanol was utilized as the extraction solvent. Upon treatment of *CASPS9* with the methanolic extracts, the expression of the genes was progressively upregulated, thus, dose-dependently increasing the rate of apoptosis. On the other hand, the expressions of *5-LOX*, *Bcl2*, and *Bcl-xL* were variably downregulated in a dose-dependent manner. This is a unique novel study that evaluated the effects of AS methanolic extracts in vitro on CRC cell lines using different dosage concentrations. We, therefore, recommend the utilization of AS and the application of methanol as the extraction solvent of choice for maximum modulatory benefits in CRC management. In addition, we suggest research on the specific metabolites in AS involved in the modulatory pathways that suppress the development of CRC and potential metastases.

## 1. Introduction

### 1.1. Background Information on Colon Cancer and Prevention Approaches

In the developed world, colon cancer is currently the third leading cause of cancer-related fatalities [1]. In general, cancer is the second leading cause of death, behind cardiovascular disorders [2]. The pathophysiology of colon tumors is very complex and poorly understood. On the other hand, the process of its onset has been linked to the interactions between risk factors such as lifestyle, inheritance, and environmental factors, among other identified causes [3,4]. Understanding the processes that swiftly proliferating malignant cells utilize to regulate their metabolism can help scientists create more effective cancer treatments [5].

To investigate and identify efficient bioactive compounds that can destroy malignant cells without harming or killing healthy cells has a huge impact in human medicine [6]. Due to this, management utilizing plant-based dietary supplements is beginning to receive attention as the most efficient way to lower the burden of colon-cancer-associated mortality [7]. Organic components found in plants may offer a fresh source for cancer treatment with a sufficiently revolutionary method of action [8]. Plant extracts have been shown to exhibit astounding therapeutic activities to treat a variety of infectious diseases, in contrast to synthetic pharmaceuticals, which are frequently observed as being associated with serious drawbacks [9].

Because of Africa’s diversity and abundance of aloe species, these plants are commonly used as a source of phytotherapeutic medication to improve human health and welfare. There are 500 species of *Aloe* L. (Asphodeloideae), a genus of flowering succulents that includes trees, shrubs, and perennials [10]. The largest genus in the Asphodelaceae family, *Aloe*, has over 400 species that range in size from tiny shrubs to enormous trees and are distributed throughout dry regions of Africa, India, and other places [11]. In South Africa, there is the greatest variety of *Aloe*. The two primary ingredients are *Aloe* exudates and *Aloe* leaf gel. Exudates come from the inner epidermal layers, while parenchymatous cells are the source of the gel. The exudates are primarily a combination of phenolic chemicals, whereas the gel is primarily composed of polysaccharides [12]. In nations such as Ethiopia, Sudan, Kenya, and Tanzania, wide grasslands and bushlands are home to numerous AS (Asphodelaceae) bushes. *Aloe engleri*, *Aloe marsabitensis*, and *Aloe floramaculata* are all synonyms of AS [12]. In our previous research [10], we postulated the potential effects of *A. secundiflora’s* active metabolites in colorectal cancer management. In the current study, we substantiate the claims postulated using specific genes present in human cells.

### 1.2. Phytoconstituent Biomolecules Present in A. secundiflora

Studies on the phytochemical and pharmacological activities of *Aloe* species have led to the discovery of various active compounds. In past decades, herbalists have used them to treat a range of diseases [11]. Most of the biologically active substances found in *Aloe* originate as anthraquinones naturally [13], as we have previously reported [10]. Terpenes, flavonoids, and tannins have been identified in the leaves (Table 1), according to preliminary phytochemical analysis [14], while naphthoquinones have been identified in the roots [15]. Anthraquinones, which are structurally related to anthracene [16], are primarily composed of Anthracenedione (9,10-anthracenedione). They are sometimes referred to as 9,10-dioxoanthracene. Anthraquinones typically occur in their glycosidic state [10]. These elements constitute the pigmentation that gives plants their hue, and they are commonly employed as natural dyes [16]. Aloin (AL) is employed in pharmacotherapy for a variety of purposes, one of which is as a laxative [17]. In both in vivo and in vitro experimental settings, AL was shown to be beneficial in lowering tumor angiogenesis and development by blocking STAT3 activation in CRC cells [18].

In the current study, we demonstrate the activity of AS in regulating the expression of *CASPS9*, *5-LOX*, *Bcl2*, *Bcl-xL*, and *COX-2* genes associated with colon carcinogenesis. In addition, *HPRT1* was employed as a housing keeping gene for internal control. *CASPS9*, the primary enzyme in the mitochondrial caspase pathway, plays a critical role in mediating apoptosis control [22,23]. The relationship between CASPS9 and CRC is currently undetermined in a tangible and substantial manner. Its correlation with clinicopathological characteristics and longevity may provide insightful data that can be used to estimate survival and choose additional treatment options [24]. While the exact sequence of events relating cancer development to *5-LOX* gene expression is equally unknown, it is clear that *5-LOX* expression is occasionally increased during neoplastic transformation [25]. *LOX* inhibitors decrease cancer cell proliferation in both in vivo and in vitro experiments and cause death through mitochondrial induction [26,27]. Targeted modulatory strategies call for knowledge of *CASPS9*, *5-LOX*, *Bcl2*, *Bcl-xL*, and *COX-2* for effective CRC medical intervention. This is a unique novel study that evaluated the effects of AS methanolic extracts in vitro on CRC cell lines at different dosage/concentration levels. We do, however, support additional research into the specific AS metabolites implicated in the modulatory pathways that prevent the growth of CRC and potential metastases.

## 2. Materials and Methods

### 2.1. Colorectal Cancer Cell Lines (Caco-2 Cell Lines)

The Caco-2 cell lines were purchased from American Type Culture Collection (ATCC) and directly delivered to our laboratory (Department of Public Health) by the University of Pecs’ Department of Biochemistry and Medical Chemistry. This type of cell line is ideally suited to be employed in studies on cancer and cytotoxicity and serves as a superb transfection carrier. In conformance with the supplier’s guidelines, the Caco-2 cell lines were preserved until use [28].

### 2.2. Plant Extract Extraction Using Methanol and Region of Acquisition

Shade-dried leaves were pulverized into a fine powder. Methanol was used in the serial exhaustive extraction (SEE). One thousand grams of AS plant organs were placed in a flask and extracted for 3 days with methanol with frequent shaking to adequately extract phytoconstituents. The crude solvent extracts were dechlorophyllated after being filtered via Whatman filter paper with varying pore diameters (Nos. 4 and 1). This technique was performed three times until all soluble elements were extracted completely [29]. The filtrate was concentrated using a rotavapor apparatus paired to a vacuum pump, a condenser apparatus to recover the solvent, and a round-bottomed flask at a temperature of 50 °C. In order to allow the samples to air dry, the concentrated solution was placed in small glass universal bottles and covered with perforated aluminum foil [12]. The dried solvent-free metabolites were stored in tightly sealed sample bottles with parafilm tape in a desiccator at 4 °C in a fridge until use [30]. The identified plant organs were collected from Kampi ya Moto in Rongai constituency, Nakuru County, Kenya and located at 0.1244° S, 35.9431° E, GPS coordinates. The annual temperature in the district is 18.75 °C (65.75 °F), which is −3.75% lower than the national average for Kenya. Furthermore, 118.62 mm (4.67 inches) of precipitation and 221.53 days of rain are typical yearly totals for Kampi Ya Moto.

### 2.3. Dissipation of Plant Extracts in DMSO

Dimethyl sulfoxide (DMSO) is a multipurpose solvent that is used in toxicology and pharmacology to improve medication delivery, dissolve various pharmaceuticals, and dissolve plant extracts [31]. It served as a suspending medium for water-insoluble crude plant extracts, as well as an inert diluent. A stock solution of 30 mg/mL was created using 0.5% DMSO and double-distilled phosphate buffer saline (ddPBS) as the dissolving and diluent solvents. The final Caco-2 cell line concentrations of 2 mg/mL, 1 mg/mL, and 0.5 mg/mL were then made using the stock solution.

### 2.4. Passaging Caco-2 Cell Lines for Bioassays

The lamina hood, which was kept sterile, was carefully placed over the petri dish or flask holding the Caco-2 cells. The utilized medium was sucked out after the dish or flask was opened. PBS was used twice to wash it. After pouring PBS-EDTA over the area, it was set aside for a while. It was then cautiously and softly sucked. To separate and detach the Caco-2 cells from clumps and the surface, respectively, 2 mL of trypsin was applied. Trypsin was applied to the surface by gently swiping the dish side to side across it. For five minutes, the dish was placed in the thermostat. The dish was removed after 5 min, and following a successful visible detachment, the Caco-2 medium was carefully added to it. The Caco-2 cells are adherent and have a propensity to stick to surfaces. The dish’s whole contents were sucked into a tube and centrifuged for five minutes at 125 rpm. The Caco-2 cells stayed at the tube’s bottom as the supernatant was sucked away. A pipette was used to gently disrupt the cells by sucking up and down while adding fresh media into the tube. The suspension was divided into fresh dishes, the medium was added, and the thermostat for growth was then turned on. The confluence was watched until it reached 70–80% so that it could be treated.

### 2.5. Exposure of Caco-2 Cell Lines to Solvent Extracts

Various quantities of the extract solutions (0.5 mg/mL, 1 mg/mL, and 2 mg/mL) were applied to 200 μL of passed Caco-2 cell lines that were refilled with new Caco-2 medium. Following that, the treated cells were incubated at 37 °C for 36 h. The condition of the cells was examined under a light microscope following the incubation period. The typical cell-doubling time of cancer cell lines, which is between 36 and 48 h, was used for the estimation of the duration of the exposure (36 h). Treatments were administered to the cells at concentrations ranging from 0.5 mg/mL at the lowest point to 2 mg/mL at the highest point to test the cells’ dose and time responsiveness. This allowed us to identify and analyze the pharmacological modulatory effects of the treatments with the greatest accuracy. The growth and potential biological impacts of exposure were assessed every 12 h.

### 2.6. Isolation of RNA

The media was removed from the cell cultures, trypsin-EDTA was applied, and they were then washed twice with PBS. After centrifuging, the cell suspension was pipetted into a 4 cm^3^ centrifuge tube. ExtraZol Tri-reagent solution diluted to 1 cm^3^ was added and allowed to sit at room temperature for 5 min. Chloroform 0.2 cm^3^ was added. The sample was centrifuged at 12,000× *g* for 10 min at 2–8 °C after a 2–3 min incubation period. A clean tube was used to transfer the aqueous phase. Isopropyl alcohol (0.2 cm^3^) was added. The sample was centrifuged once more at 12,000× *g* for 10 min at 2–8 °C after being incubated for 10 min.

The RNA precipitate is frequently invisible prior to centrifugation but produces a gel-like pellet at the tube’s bottom following centrifugation. The supernatant was removed before washing the RNA pellet with 1 cm^3^ of 75% alcohol. The pellet was then centrifuged at 7500× *g* for 5 min at 2–8 °C after vortexing. The pellet was dried after the supernatant was drained off. It was then dissolved in 50–100 L of DEPC water, which is RN-ase free. After being vortexed, the sample was incubated for 10 min at 55 °C. Before usage, the isolated total RNA was kept at −80 °C.

### 2.7. Protocol and Equipment Used for qRT-PCR (SYBR Green Protocol)

Nucleic acid quantification is sensitive, specific, and repeatable with real-time quantitative PCR. According to the manufacturer’s instructions, one-step PCR was carried out using the One-Step Detect SyGreen Lo-ROX one-step RT-PCR kit (Nucleotest Bio Ltd. PB25.11–12) on a 96-well plate using a LightCycler 480 qPCR platform. The thermal program was set up as follows: 45 cycles of 95 °C for 5 s, 56 °C for 15 s, and 72 °C for 5 s, with a fluorescence readout being obtained at the conclusion of each cycle. The sample was incubated at 42 °C for 5 min, followed by incubation at 95 °C for 3 min. A melting curve analysis (95 °C—5 s, 65 °C—60 s, 97 °C) was performed after each run to validate amplification specificity. The reaction mixture was as follows: 10 l Master Mix, 0.4 l RT Mix, 0.4 l dUTP, 0.4 l primers, and 5 l mRNA template added to a total volume of 20 l of sterile double-distilled water. Integrated DNA Technologies (Bio-Sciences) created the primers, and Primer Express™ Software v3.0.1 was used to create the sequences (Table 2).

### 2.8. qRT-PCR Result Analysis

The Cp numbers used to express the PCR findings represent the intersection of the amplification curve and the threshold value. Using the 2-Cp (Livak method) and the Cp values, the fold changes of the target genes from the control sample were calculated [32].

### 2.9. Data Analysis

IBM SPSS 26.0 (IBM Corp. Released 2019. IBM SPSS Statistics for Windows, Version 26.0. Armonk, NY, USA: IBM Corp) and MS Excel (Microsoft Corp., released in 2013, Redmond, WA, USA) were used to calculate the statistical analysis. IBM SPSS Statistics for Windows, version 26.0.3. From the collected data, the normality analysis was conducted using the Kolmogorov–Smirnov test, and the means of the relevant variables were then compared using the ANOVA test. If the results were significant at the 95% confidence level, the p value was *p* ≤ 0.05.

## 3. Results

### 3.1. Upregulatory Effects of AS Extracts on CASPS9 Expression

**Figure 1:**
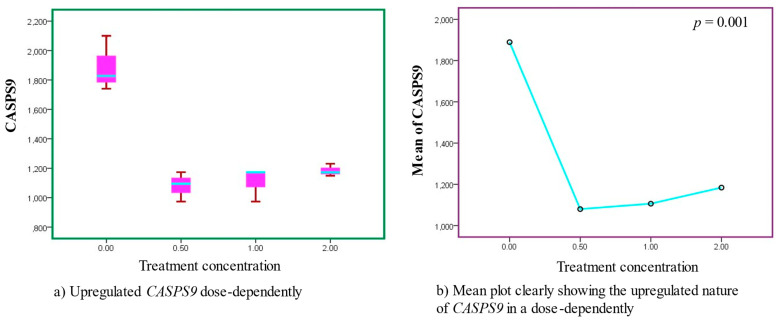
Gradual upregulatory activities were observed in *CASPS9* after treatment with AS leaf extracts, as clearly shown in sub-figures (**a**,**b**). The regulatory characteristics were statistically significant.

When the *CASPS9* genes were treated with AS’s leaf extracts, apoptosis was observed to gradually occur upon exposure. Of interest, the increased optimal activity of apoptotic expressions was observed at a dosage concentration of 1 mg/mL. However, the apoptotic activity diminished and was at minimum in a concentration of 2 mg/mL. Observably, the expressions of *CASPS9* were progressively upregulated in a dose-dependent manner on exposure to the treatment extracts. In addition, the effects were statistically significant (*p* < 0.001), (Figure 1, Table 3 and Table 4).

### 3.2. Downregulatory Effects of AS Extracts on 5-LOX Expression

**Figure 2:**
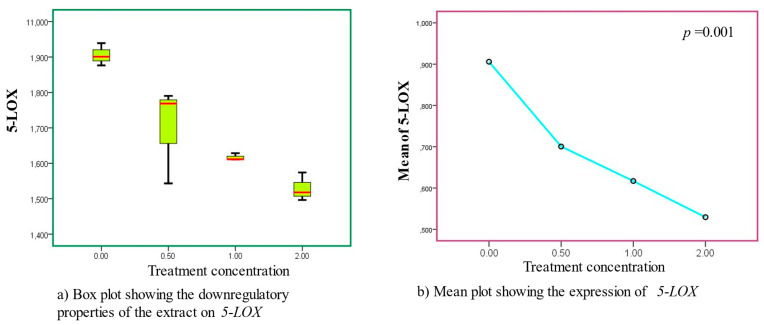
Downregulatory effects were observed in *5-LOX* upon treatment with AS leaf extracts, as clearly shown in sub-figures (**a**,**b**). The modulatory expressions were statistically significant. (**a**) Box plot, (**b**) mean plot.

After the *5-LOX* genes were treated with AS extract, a characteristic decrease in expression was gradually observed across the varying concentrations of exposure. An optimal mechanism of action resulting in a high decrease in expression was seen to occur at a concentration of 0.5 mg/mL. While the mechanism of action steadily increased at a dose of 2 mg/mL, very little transcriptional activity was seen at a concentration of 1 mg/mL. It is appropriate to state that the downregulatory effects in this investigation were dose dependent. The analysis yielded statistically significant findings (Figure 2, Table 3 and Table 4).

### 3.3. Downregulatory Effects of AS Extracts on Bcl2 Expressions

**Figure 3:**
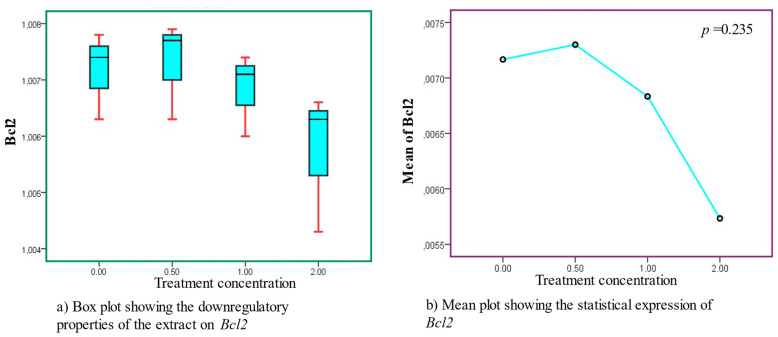
Downregulatory properties recorded in *Bcl2* after treatment with AS leaf extracts. Sub-figures (**a**,**b**) represent the box and mean lots, respectively.

In all treatment concentrations, the decreased expression of *Bcl2* was observed to be dose-dependent. Even though the statistical output was not statistically significant (*p* = 0.235), the extract treatments exhibited significant downregulatory effects required to stimulate inhibitory cellular growth effects, as shown in Figure 3, Table 3 and Table 4.

### 3.4. Downregulatory Effects of AS Extracts on Bcl-xL Gene Expressions

**Figure 4:**
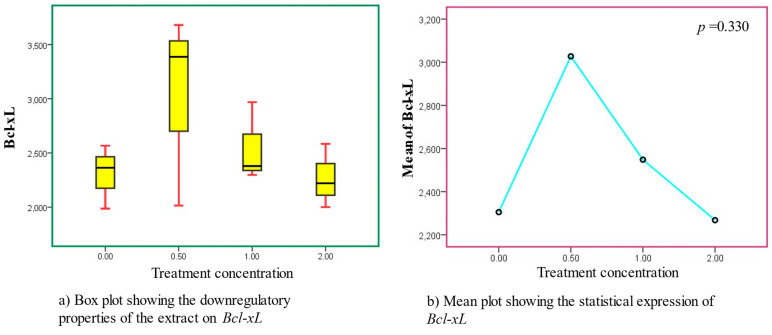
Downregulatory properties recorded in *Bcl-xL* after treatment with AS leaf extracts. Sub-figures (**a**,**b**) represent the box and mean lots, respectively.

The downregulatory effects were progressively dose-dependent with optimal activities observed at 0.50 mg/mL (Figure 4). The treatments exhibited significant modulatory and beneficial properties as required to stimulate downregulation. The downregulatory effects were sufficient to elicit beneficial inhibitive properties (Table 3 and Table 4).

### 3.5. Downregulatory Effects of AS Extracts on COX-2 Expression at High Concentrations

**Figure 5:**
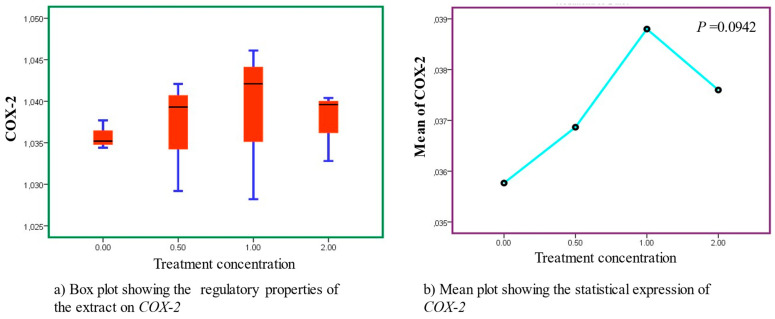
Varied regulatory properties observed in *COX-2* after treatment with AS leaf extracts. Sub-figures (**a**,**b**) represent the box and mean lots, respectively.

**Figure 6:**
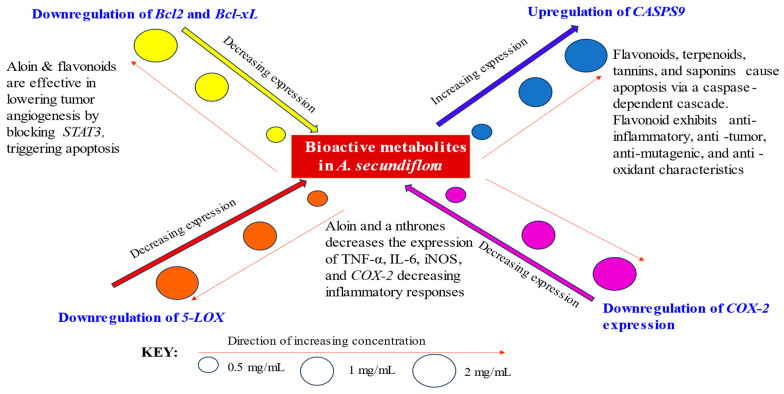
A summarized mechanistic schematic presentation of *CASPS9*, *5-LOX*, *Bcl2/Bcl-xL*, and *COX-2* relative expressions with increasing AS extract concentration.

When the Caco-2 cell lines were treated with methanolic leaf extracts, the *COX-2* genes were modulated variably with an increasing dosage across the concentration gradients. Notably, at a low concentration, the extract products stimulated upregulation of *COX-2* genome expression. However, downregulatory effects were observed at high concentrations (2.0 mg/mL), indicating potential optimal benefits at a high dosage concentration, as exhibited in (Figure 5). In summary, the concentration of AS extract treatment poses a direct impact on molecular activities, resulting in relative changes in gene expression (Figure 6, Table 3 and Table 4).

## 4. Discussion

### 4.1. Phytotherapeutic Effects of AS Leaf Extracts on CASPS9 Expression

An essential mediator in the regulation of apoptosis is *CASPS9*, the starter of the mitochondrial caspase cascade. These enzymes (Caspases) are a component of a cascade that is triggered by pro-apoptotic cues and causes the fragmentation of cells, as well as the dissociation of many peptides. For the aim of medical treatment, it is essential to selectively modulate apoptosis [22,23]. In a variety of biological events, apoptosis is a crucial physiological process that involves the selective elimination of cells [33]. According to some of the research, inhibiting spontaneous apoptosis raises the risk of developing cancer [34,35]. Similarly, it has been noted that a lower rate of apoptosis is strongly associated with a higher frequency of colorectal adenoma [36]. Aloin was shown to significantly decrease tumor sizes and weight in mice xenografts while inducing apoptosis and lowering tumor cell viability in vitro [18]. It was demonstrated that natural plant-based constituents found in AS, such as flavonoids [37], terpenoids, tannins [38], and saponins [39,40,41], can cause and enhance apoptosis via a caspase-dependent cascade. In addition to their powerful anti-inflammatory, anti-tumor, anti-mutagenic, and antioxidant characteristics, flavonoids also have the capacity to regulate key cellular enzyme functions [42]. The findings of this study showed that methanolic extracts demonstrated high efficacy with significant activity in the upregulation of *CASPS9* in a dose-dependent manner. The increased upregulation of *CASPS9* resulted in decreased tumor development and, thus, an important breakthrough in CRC growth inhibition. The capacity to cause apoptosis in gastrointestinal epithelial cells is one potential chemo-prevention technique [35]. Cancer incidence has been postulated to increase when spontaneous apoptosis is suppressed [34,35]. Similar findings have been made regarding the relationship between colorectal adenoma prevalence and apoptosis [36]. One of the prospective chemoprevention techniques is the ability to cause apoptosis in epithelial cells with gastrointestinal origin [35]. For this reason, a promising direction for colorectal cancer research is the study of the apoptotic process. Therefore, the level of expression of the apoptosis-associated *CASPS9* genes may be helpful in predicting the prognosis for people with stage II colorectal cancer. From our findings, methanol is, thus, highly recommended for use as an efficient extraction solvent for bioactive ingredients in AS with demonstrated upregulatory effects of *CASPS9* in CRC treatment. Of great significance, the dose-dependent variation of these genes under the influence of AS leaf extracts has not been investigated in the previous literature. Thus, highlighting the importance of this research, which evaluates and documents the activity of *A. secundiflora’s* active metabolites in modulation of the CRC *CASPS9* genes responsible for programmed cell death (apoptosis), for deployment in CRC treatment.

### 4.2. Phytotherapeutic Effects of AS Leaf Extracts on 5-LOX Expression

Inhibiting the expression of *5-LOX*, which is upregulated in colorectal cancer, could be helpful in both the prevention and therapy of the disease [43]. It has been demonstrated that when arachidonic acid, a polyunsaturated fatty acid, is metabolized by either the COX pathway or the LOX mechanism, eicosanoids such as prostaglandins, thromboxanes, and leukotrienes, among others, operate as potent autocrine and paracrine regulators of cell biology. Only a few of the physiological and pathological responses that these compounds are known to alter include the growth and invasiveness of tumor cells, as well as the suppression of immune surveillance [44]. Targeting arachidonic acid pathways may be useful in delaying the progression of CRC and other types of malignancies, since LOXs enzymes produce metabolites in the arachidonic acid pathway that seem to promote carcinogenesis.

The results of this study demonstrated that downregulatory properties were observed to occur in a dose-dependent manner, with the expressions of *5-LOX* being suppressed with increasing concentration. Although prostaglandins (PGs) and other COX-derived metabolites have received most of the attention, the new research indicates that leukotrienes (LTs) and hydroxyeicosatetraenoic acids (HETEs), two products catalyzed by LOX enzymes, also have a significant biological impact on the initiation and progression of human cancers. In several human cancer cell lines and tissues, including those of the colon [43,45,46], an increase in the expression of *5-LOX* and their metabolites has been found. This over-expression was reported to be significantly linked to tumor cell proliferation, resistance to apoptosis, and angiogenesis [47,48]. Additionally, it was discovered that the direct suppression of *5-LOX* or *12-LOX* significantly reduced the development of tumor cells [44,47,48,49].

Our most important finding was the substantial correlation between extract dose concentration and *5-LOX* expression level. This finding is consistent with other research, which showed that gastric or colorectal [47] tumors with higher *5-LOX* and *COX-2* levels grew deeper and larger. Additionally, it lends credence to the idea that *5-LOX* and *COX-2* share features of expression and function that are proangiogenic and anti-apoptotic [50] as well as substrate preference in human cancer. Like COX-2 and PGs, 5-LOX enzymes and their products may operate on tumor cells by inhibiting apoptosis, increasing cell proliferation, and stimulating angiogenesis, according to a number of experimental investigations [48,50]. The degree of *5-LOX* expression and LTB4 synthesis in cancer cell lines was recently found to be correlated with the dose- and time-dependent reduction in cell viability and induction of apoptosis caused by *5-LOX* inhibitors [48]. A key mechanism of 5-*LOX* activities on proliferation and apoptosis was the release of endothelial growth factor (VEGF) and mRNA levels by *5-LOX* activity in malignant mesothelial cells [48].

The abundance of biomolecules found in AS, as we have previously described [10], suggests the value of using this plant as a potential anti-CRC agent with the assurance of phytotherapeutic effects. The overwhelming evidence is in congruent support of our findings with regards to the benefits of suppressing the expression of *5-LOX*. *A. secundiflora* (AS) is a promising phytotherapeutic plant, and based on our thorough research, this is among the first studies to assess the levels of expression and clinicopathologic significance of the *5-LOX* gene on human CRC using AS. AS leaf extracts are, therefore, unreservedly perfect natural *5-LOX* inhibitors whose exploitation for therapy is of paramount importance in CRC management. Thus, our findings imply that *5-LOX* overexpression may have a significant impact on the emergence of CRC. Therefore, blocking this metabolic pathway might be a timely and useful therapeutic strategy for both the prevention and treatment of CRC.

### 4.3. Phytotherapeutic Effects of AS Leaf Extracts on Bcl2 and Bcl-xL Expression

The best-defined protein family involved in the regulation of apoptotic cell death is the Bcl-2 protein family, which comprises members that are both anti- and pro-apoptotic. The anti-apoptotic members of this family include, among others, *Bcl2* and *Bcl-xL*. In the present study, it was exclusively demonstrated that in all treatments, the downregulatory effects of the extracts on *Bcl-xL* and *Bcl2* were progressively influenced dose-dependently. All of the extract treatments exhibited significant downregulatory and beneficial properties as required to stimulate inhibitory properties on genome expression. Other investigators have also asserted that aloin is effective in lowering tumor angiogenesis and growth by blocking *STAT3* activation in CRC cells, which, in turn, controls the expression of the antiapoptotic protein *Bcl-xL* gene [18].

One of the latent self-signaling transcription factors in the cytoplasm is *STAT3* (e.g., VEGF), which is activated by cytokines (such as IL-6) and progenitor cells. The stimulation of STAT3 homodimerization and nuclear translocation modulates the transcription of responsive genes encoding apoptotic-cell-death inhibitors (e.g., *Bcl-xL*, *Bcl2*) and inducers of angiogenesis (e.g., VEGF) [51]. These genes play roles in human defense evasion, angiogenesis, metastatic spread, cell survival, differentiation, and programmed cell death [52]. In recent years, there has been an abundance of research demonstrating that blocking constitutive STAT3 signaling substantially inhibits tumor development and triggers apoptosis [51,53]. By downregulating the biosynthesis of *Bcl2* and *Bcl-xL* through the p53 signaling pathway, a notable apoptotic target in many cancer types, flavonoids diminish a significant dysregulated pathway in cancer [54]. Therefore, it is crucial to realize that Bcl2 and Bcl-xL suppression causes advantageous apoptotic effects, which subsequently reduce CRC cancer growth. Pharmacological manipulation of the Bcl-2 family activities will be limited until a deeper scientific knowledge of how Bcl-2 family proteins control apoptosis in cells is established [55]. This is, therefore, the first study that has successfully evaluated the downregulatory effects of *A. secundiflora’s* extracts on the expression of *Bcl2* and *Bcl-xL* in colorectal cancer cell lines. In line with these fundamental findings, the methanolic leaf extracts of AS are recommended for considerable deployment for further in vivo and subsequent clinical trials for therapeutic management of CRC in humans, with substantial beneficial effects postulated.

### 4.4. Phytotherapeutic Effects of AS Leaf Extracts on COX-2 Expression

With regards to *COX-2* expression, cancer risk is increased by persistent inflammation [56], while strong induction of *COX-2* occurs during inflammation. This study demonstrates that downregulatory effects were quite minimal across all doses applied but sufficient to elicit beneficial properties. Of significance to note is that one of the crucial enzymes involved in the manufacture of prostaglandins, which is linked to inflammatory processes, is the COX-2 enzyme. Relief from these inflammatory conditions may result from inhibition of the COX-2 enzyme, and consequently, of the prostaglandin biosynthetic pathway [57]. Tumor necrosis factor alpha (TNF-α), interleukin 6 (IL-6), inducible nitric oxide synthase (iNOS), and cyclooxygenase-2 (COX-2) expression are all downregulated by aloin (highly abundant in the *Aloe* species), which has been shown to suppress lipopolysaccharide-induced pro-inflammatory cytokine secretion and nitric oxide production [58]. Phenols such as anthrones that are also abundant in AS were reported to strongly inhibit the expression of *COX-2* [59]. Aloin and aloe-emodin are said to limit the expression of iNOS and COX-2 mRNA, hence, reducing inflammatory responses. The authors further assert that aloe-emodin may be a crucial component behind aloe’s anti-inflammatory properties [60]. The phenolic components of the methanolic extract may have contributed to the downregulatory effects of the extracts. Therefore, it was presupposed that the radical scavenging capabilities of naphthalene analogs and the inhibition of *COX-2* by anthraquinones and naphthalene derivatives were the mechanisms by which the inhibitory properties were mediated. Remarkably, this is one of the first studies that has demonstrated an explicit link between AS leaf extracts and *COX-2* downregulation in CRC cell lines, with prospective application for CRC management.

## 5. Conclusions

In this remarkable study, CRC cell lines were exposed to various AS methanolic extract doses in vitro. In our investigation, AS’s leaf extracts were arduously proven and listed as being incredibly effective at modulating the activities of *CASPS9*, *5-LOX*, *Bcl2*, *Bcl-xL*, and *COX-2* genes for pharmacotherapeutic beneficial effects. Methanol was also demonstrated to be a promising extraction solvent for the effective metabolites responsible for the modulatory properties discussed in our study. Given the copious amounts of phytoconstituent biomolecules present in AS, it is beneficial to use the plant as a prospective CRC inhibiting agent with potent phytotherapeutic benefits. To treat CRC, targeted apoptotic modulation necessitates an understanding of genome programming. The regulatory effects exhibited in this research establish AS as an important plant of choice, one whose extracts have demonstrated significant CRC inhibitory properties. This is one of the initial studies that explicitly hyperlinks the positive effects of AS leaf extracts on the targeted genes in our study for potential applications for therapeutic management of CRC. We, therefore, strongly recommend the utilization of AS for further in vivo and subsequent clinical trials for CRC management. In addition, we suggest the application of methanol as a promising AS extraction solvent for maximum regulatory benefits towards *CASPS9*, *5-LOX*, *Bcl2*, *Bcl-xL*, and *COX-2* expression. Finally, we also encourage further investigation into the particular AS metabolites involved in the modulatory pathways that inhibit the development of CRC, as well as potential metastases.

## Figures and Tables

**Table 1:** Phytoconstituent biomolecules present in A. secundiflora [10].

Plant	Phytoconstituents Present in Roots	Phytoconstituents Present in Leaves	Ref.
*Aloe secundiflora*	Anthraquinones (Chrysophanol, Helminthosporin, Aloe-emodin, Aloesaponarin II, and Aloesaponarin I), laccaic acid D, methyl ester, and asphodelin. Naphthoquinones (5-hydroxy-3,6-dimethoxy-2-methylnaphthalene-1,4-dione and 5,8-dihydroxy-3-methoxy-2-methylnaphthalene-1,4-dione)	Phenols such as anthrones (aloenin, aloenin B, isobarbaloin, barbaloin, and other aloin derivatives), chromones and phenylpyrones, Alkaloids, Saponin, Tannins, Flavonoids (nthoxanthins, flavanones, flavanols, flavans, and anthocyanidin), Steroids, Cardiac Glycosides, Aloeresin, Anthraquinones Aloin, Hydro-xyaloins, Polyphenols, and Terpenoids	[14,15,16,19,20,21]

**Table 2:** Forward and reverse primer sequences adopted and applied in the experimental study.

Primer ID	Forward Primer	Reverse Primer
*COX-2*	CGGTGAAACTCTGGCTAGACAG	GCAAACCGTAGATGCTCAGGGA
*5-LOX*	GGAGAACCTGTTCATCAACCGC	CAGGTCTTCCTGCCAGTGATTC
*Bcl2*	ATCGCCCTGTGGATGACTGAGT	GCCAGGAGAAATCAAACAGAGGC
*Bcl-xL*	GCCACTTACCTGAATGACCACC	AACCAGCGGTTGAAGCGTTCCT
*Casp9*	GTTTGAGGACCTTCGACCAGCT	CAACGTACCAGGAGCCACTCTT
*HPRT1*	TGCTTCTCCTCAGCTTCA	CTCAGGAGGAGGAAGCC

**Table 3:** Post hoc computed test analysis of multiple relationships showing the targeted gene expressions at increasing concentrations (Tukey HSD, multiple comparisons ^a^).

Dependent Variable	(I) Conc	(J) Conc	Mean Difference (I–J)	Std. Error	Sig.	95% Confidence Interval	
						Lower Bound	Upper Bound
*COX2*	0.00	0.50	−0.0011000	0.0050780	0.996	−0.017362	0.015162
		1.00	−0.0030333	0.0050780	0.930	−0.019295	0.013228
		2.00	−0.0018333	0.0050780	0.983	−0.018095	0.014428
	0.50	0.00	0.0011000	0.0050780	0.996	−0.015162	0.017362
		1.00	−0.0019333	0.0050780	0.980	−0.018195	0.014328
		2.00	−0.0007333	0.0050780	0.999	−0.016995	0.015528
	1.00	0.00	0.0030333	0.0050780	0.930	−0.013228	0.019295
		0.50	0.0019333	0.0050780	0.980	−0.014328	0.018195
		2.00	0.0012000	0.0050780	0.995	−0.015062	0.017462
	2.00	0.00	0.0018333	0.0050780	0.983	−0.014428	0.018095
		0.50	0.0007333	0.0050780	0.999	−0.015528	0.016995
		1.00	−0.0012000	0.0050780	0.995	−0.017462	0.015062
*Lox5*	0.00	0.50	0.2052000 *	0.0596828	0.036	0.014075	0.396325
		1.00	0.2887667 *	0.0596828	0.006	0.097641	0.479892
		2.00	0.3763333 *	0.0596828	0.001	0.185208	0.567459
	0.50	0.00	−0.2052000 *	0.0596828	0.036	−0.396325	−0.014075
		1.00	0.0835667	0.0596828	0.533	−0.107559	0.274692
		2.00	0.1711333	0.0596828	0.080	−0.019992	0.362259
	1.00	0.00	−0.2887667 *	0.0596828	0.006	−0.479892	−0.097641
		0.50	−0.0835667	0.0596828	0.533	−0.274692	0.107559
		2.00	0.0875667	0.0596828	0.497	−0.103559	0.278692
	2.00	0.00	−0.3763333 *	0.0596828	0.001	−0.567459	−0.185208
		0.50	−0.1711333	0.0596828	0.080	−0.362259	0.019992
		1.00	−0.0875667	0.0596828	0.497	−0.278692	0.103559
*Bcl2*	0.00	0.50	−0.0001333	0.0007605	0.998	−0.002569	0.002302
		1.00	0.0003333	0.0007605	0.970	−0.002102	0.002769
		2.00	0.0014333	0.0007605	0.306	−0.001002	0.003869
	0.50	0.00	0.0001333	0.0007605	0.998	−0.002302	0.002569
		1.00	0.0004667	0.0007605	0.925	−0.001969	0.002902
		2.00	0.0015667	0.0007605	0.244	−0.000869	0.004002
	1.00	0.00	−0.0003333	0.0007605	0.970	−0.002769	0.002102
		0.50	−0.0004667	0.0007605	0.925	−0.002902	0.001969
		2.00	0.0011000	0.0007605	0.508	−0.001335	0.003535
	2.00	0.00	−0.0014333	0.0007605	0.306	−0.003869	0.001002
		0.50	−0.0015667	0.0007605	0.244	−0.004002	0.000869
		1.00	−0.0011000	0.0007605	0.508	−0.003535	0.001335
*Bcl-xL*	0.00	0.50	−0.7222000	0.4281958	0.389	−2.093434	0.649034
		1.00	−0.2432333	0.4281958	0.939	−1.614467	1.128000
		2.00	0.0371000	0.4281958	1.000	−1.334134	1.408334
	0.50	0.00	0.7222000	0.4281958	0.389	−0.649034	2.093434
		1.00	0.4789667	0.4281958	0.689	−0.892267	1.850200
		2.00	0.7593000	0.4281958	0.351	−0.611934	2.130534
	1.00	0.00	0.2432333	0.4281958	0.939	−1.128000	1.614467
		0.50	−0.4789667	0.4281958	0.689	−1.850200	0.892267
		2.00	0.2803333	0.4281958	0.911	−1.090900	1.651567
	2.00	0.00	−0.0371000	0.4281958	1.000	−1.408334	1.334134
		0.50	−0.7593000	0.4281958	0.351	−2.130534	0.611934
		1.00	−0.2803333	0.4281958	0.911	−1.651567	1.090900
*CASPS9*	0.00	0.50	0.8094667 *	0.1002133	0.000	.488548	1.130385
		1.00	0.7833000 *	0.1002133	0.000	.462382	1.104218
		2.00	0.7052000 *	0.1002133	0.000	.384282	1.026118
	0.50	0.00	−0.8094667 *	0.1002133	0.000	−1.130385	−0.488548
		1.00	−0.0261667	0.1002133	0.993	−0.347085	0.294752
		2.00	−0.1042667	0.1002133	0.732	−0.425185	0.216652
	1.00	0.00	−0.7833000 *	0.1002133	0.000	−1.104218	−0.462382
		0.50	0.0261667	0.1002133	0.993	−0.294752	0.347085
		2.00	−0.0781000	0.1002133	0.862	−0.399018	0.242818
	2.00	0.00	−0.7052000 *	0.1002133	0.000	−1.026118	−0.384282
		0.50	0.1042667	0.1002133	0.732	−0.216652	0.425185
		1.00	0.0781000	0.1002133	0.862	−0.242818	0.399018

* The mean difference is significant at the 0.05 level; ^a^ treatment = methanolic leaf extracts.

**Table 4:** Statistical analysis of the effects of methanolic leaf extracts on the targeted genes.

Target Genes		Sum of Squares	df	Mean Square	F	Sig.
*COX2*	Between Groups	0.000	3	0.000	0.126	0.942
	Within Groups	0.000	8	0.000		
	Total	0.000	11			
*Lox5*	Between Groups	0.233	3	0.078	14.554	0.001
	Within Groups	0.043	8	0.005		
	Total	0.276	11			
*Bcl2*	Between Groups	0.000	3	0.000	1.748	0.235
	Within Groups	0.000	8	0.000		
	Total	0.000	11			
*Bcl-xL*	Between Groups	1.100	3	0.367	1.333	0.330
	Within Groups	2.200	8	0.275		
	Total	3.300	11			
*Caspase9*	Between Groups	1.338	3	0.446	29.603	0.000
	Within Groups	0.121	8	0.015		
	Total	1.458	11			

## Data Availability

The datasets generated and/or analyzed during the current study are available from the corresponding author on reasonable request.
